# Mechanisms linking childhood trauma exposure and psychopathology: a transdiagnostic model of risk and resilience

**DOI:** 10.1186/s12916-020-01561-6

**Published:** 2020-04-01

**Authors:** Katie A. McLaughlin, Natalie L. Colich, Alexandra M. Rodman, David G. Weissman

**Affiliations:** 1 Department of Psychology, Harvard University, 33 Kirkland Street, Cambridge, MA 02138 USA; 2 Department of Psychology, University of Washington, Box 351525, Seattle, WA 98195 USA

**Keywords:** Childhood trauma, Psychopathology, Transdiagnostic, Social information processing, Emotion regulation, Emotional processing, Accelerated aging, Biological aging

## Abstract

**Background:**

Transdiagnostic processes confer risk for multiple types of psychopathology and explain the co-occurrence of different disorders. For this reason, transdiagnostic processes provide ideal targets for early intervention and treatment. Childhood trauma exposure is associated with elevated risk for virtually all commonly occurring forms of psychopathology. We articulate a transdiagnostic model of the developmental mechanisms that explain the strong links between childhood trauma and psychopathology as well as protective factors that promote resilience against multiple forms of psychopathology.

**Main body:**

We present a model of transdiagnostic mechanisms spanning three broad domains: social information processing, emotional processing, and accelerated biological aging. Changes in social information processing that prioritize threat-related information—such as heightened perceptual sensitivity to threat, misclassification of negative and neutral emotions as anger, and attention biases towards threat-related cues—have been consistently observed in children who have experienced trauma. Patterns of emotional processing common in children exposed to trauma include elevated emotional reactivity to threat-related stimuli, low emotional awareness, and difficulties with emotional learning and emotion regulation. More recently, a pattern of accelerated aging across multiple biological metrics, including pubertal development and cellular aging, has been found in trauma-exposed children. Although these changes in social information processing, emotional responding, and the pace of biological aging reflect developmental adaptations that may promote safety and provide other benefits for children raised in dangerous environments, they have been consistently associated with the emergence of multiple forms of internalizing and externalizing psychopathology and explain the link between childhood trauma exposure and transdiagnostic psychopathology. Children with higher levels of social support, particularly from caregivers, are less likely to develop psychopathology following trauma exposure. Caregiver buffering of threat-related processing may be one mechanism explaining this protective effect.

**Conclusion:**

Childhood trauma exposure is a powerful transdiagnostic risk factor associated with elevated risk for multiple forms of psychopathology across development. Changes in threat-related social and emotional processing and accelerated biological aging serve as transdiagnostic mechanisms linking childhood trauma with psychopathology. These transdiagnostic mechanisms represent critical targets for early interventions aimed at preventing the emergence of psychopathology in children who have experienced trauma.

## Background

**Fig. 1:**
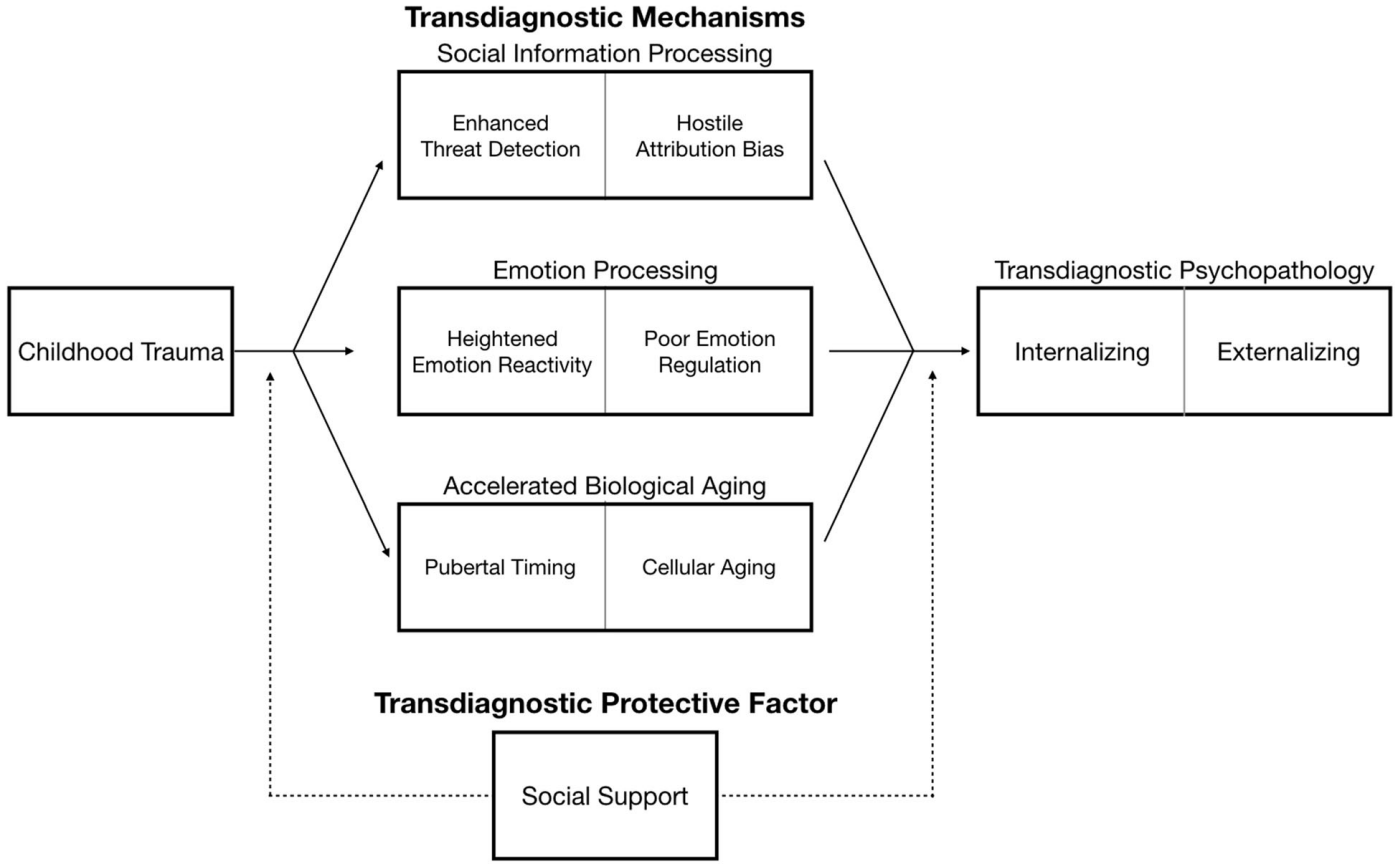
A transdiagnostic model of mechanisms linking childhood trauma to psychopathology spanning social, emotional, and biological domains. Solid lines represent direct associations between childhood trauma and social information processing, emotion processing, and biological aging as mechanisms of risk for both internalizing and externalizing psychopathology. Dashed line depicts the protective role social support plays in buffering those with history of childhood trauma from developing transdiagnostic psychopathology

A central tenet of developmental approaches to psychopathology is that the same environmental experience, psychological process, or neurobiological factor may ultimately lead to different developmental outcomes or forms of psychopathology across people, a phenomenon known as multifinality [1]. Increasing recognition of this multifinality has led to the emergence of transdiagnostic models of psychopathology. Transdiagnostic approaches seek to identify core psychological and neurobiological processes that underlie multiple forms of psychopathology [2]. These models afford many benefits over disorder-specific approaches by identifying core mechanisms that might play a role in many different forms of psychopathology, determining whether such factors explain the high rates of comorbidity across disorders, and providing key targets for interventions that could be used to prevent or treat multiple types of psychopathology [2]. Much existing work on transdiagnostic factors has focused on cognitive and affective processes that are associated with vulnerability for multiple forms of psychopathology, such as rumination and other forms of repetitive negative thinking or disruptions in emotion regulation [3–5]. Here, we expand this focus to encompass environmental experiences occurring early in development. Specifically, we argue that exposure to trauma in childhood is a particularly powerful transdiagnostic factor that is associated with increased risk for many types of psychopathology. Next, we present a transdiagnostic model that highlights several key mechanisms that appear to explain how childhood trauma ultimately confers risk for multiple forms of psychopathology throughout the life-course (see Fig. 1). We end by reviewing the implications of this transdiagnostic model for screening and early intervention.

## Childhood trauma exposure

Traumatic events are experiences that have a high potential for harm, including actual or threatened death, serious injury, or sexual violation [6]. We focus here on traumatic events that involve exposure to interpersonal violence—including experiences of direct victimization as well as witnessing violence occurring to another person, as these types of traumatic events have particularly strong associations with psychopathology [7–9].

A majority of children and adolescents in the United States will experience a traumatic event by the time they reach adulthood [9]. A smaller but still substantial proportion of children—about one quarter to one third across studies—have experienced or witnessed interpersonal violence [9–11]. Childhood trauma exposure is also a common problem cross-nationally [10, 12, 13].

Exposure to childhood trauma is a potent risk factor for multiple forms of psychopathology. Evidence from population-based studies suggests that children who have experienced trauma are about twice as likely to develop a mental disorder compared to those who have never experienced trauma [10, 14]. This elevated risk for the emergence of psychopathology is present not only during childhood but persists throughout adolescence and adulthood [15–17]. Exposure to childhood trauma is associated with virtually all commonly occurring forms of psychopathology, including mood, anxiety, substance use, and disruptive behavior disorders, with little meaningful variation in the strength of associations across disorders [14, 16–20]. Childhood trauma is also associated with psychotic experiences [21, 22] and suicidal ideation and attempts [23–26]. In addition, more than one in six children exposed to trauma develop post-traumatic stress disorder (PTSD) [27], a diagnosis that occurs only in those who have experienced a traumatic event. Together, these findings demonstrate clearly that childhood trauma is associated with increased risk for psychopathology transdiagnostically.

Several population-based studies have examined whether childhood trauma has stronger associations with some forms of psychopathology than others. These studies have repeatedly shown the associations of child maltreatment—which includes traumatic experiences of abuse as well those involving neglect—with lifetime psychopathology operate largely through a latent factor for internalizing and externalizing psychopathology, with no significant direct effects on specific mental disorders after adjusting for associations with this latent factor [28, 29]. This pattern suggests that exposure to trauma in childhood likely influences the development of psychological and neurobiological processes that confer broad vulnerability to multiple types of psychopathology. In the following sections, we describe three sets of potential mechanisms that appear to underlie the link between childhood trauma and psychopathology transdiagnostically.

## Transdiagnostic mechanisms

Trauma exposure may have a particularly pernicious influence when it happens early in life given the heightened brain plasticity that characterizes childhood and adolescence [30]. This plasticity reflects the ability of the brain to change in response to environmental experiences. Although elevated brain plasticity in early life confers many advantages by allowing children to learn rapidly from experience and adapt to the environment in which they are being raised, it may also have long-term costs for children raised in environments that are dangerous. Specifically, environments characterized by violence and high potential for harm can influence patterns of social, emotional, and neurobiological development in ways that facilitate the rapid detection of potential threats [31]. Although these developmental adaptations may enhance safety in dangerous environments by mobilizing behavioral responses to avoid threats, they also may increase risk for multiple forms of psychopathology. To serve as a transdiagnostic mechanism, a particular process must (a) be influenced by childhood trauma exposure, (b) predict the later emergence of multiple forms of psychopathology, and (c) explain the association between childhood trauma and later psychopathology.

Importantly, the mechanisms we highlight below appear to be relatively specific to experiences of childhood trauma as opposed to adversity more globally. For example, none of these mechanisms have been consistently observed in children who experience forms of adversity involving deprivation (e.g., neglect). Indeed, recent conceptual models argue that different types of adverse early-life environments have distinct influences on cognitive, emotional, and neurobiological development, in ways that reflect plasticity mechanisms that allow children to adapt to the environments in which they are developing [32–34]. Below, we outline several fundamental processes that reflect these types of developmental adaptations to threatening early environments but also appear to confer risk for psychopathology transdiagnostically (see Fig. 1).

### Social information processing mechanisms

Biases in social information processing—specifically, the perception, identification, and interpretation of social cues—are one set of core mechanisms that contribute to the strong association between childhood trauma and transdiagnostic psychopathology [31, 35]. Across a range of social information processing domains, children who have experienced trauma exhibit biases that prioritize the identification of potentially threatening social cues and are more likely to perceive or classify such cues as threatening [36–38].

Children who have experienced trauma can identify expressions of anger or fear with less perceptual information than children who have never experienced trauma; this heightened perceptual sensitivity is specific to threat cues and does not exist for other emotions (e.g., happiness and sadness) [37–39]. The magnitude of perceptual sensitivity to threat increases as the severity of trauma increases [40] and persists into adulthood following trauma exposure in childhood [41]. Similar patterns have been observed using tasks that assess attentional processes involved in orienting towards and disengaging from emotionally salient stimuli. Children who have experienced trauma exhibit faster attentional orienting to angry facial expressions and vocal cues, but not other emotions, suggesting that their attention is more easily captured by threatening stimuli than children who have never experienced trauma [42–44]. Once their attention has been captured, children with trauma histories also have more difficulty disengaging from anger cues than children who have not experienced trauma [45]. Finally, trauma-exposed children appear to use more liberal criteria for classifying emotional expressions and social situations as threatening (i.e., involving anger); specifically, they are more likely than children who have never experienced trauma to misclassify other negative emotions like sadness and fear and even neutral facial expressions as anger [37, 38]. Importantly, children who have experienced neglect have difficulty discriminating between different emotions broadly but do not exhibit these types of threat-related biases in perceptual sensitivity or emotion identification [37].

These social information processing biases extend beyond facial and vocal expressions to the interpretation of a broader range of social situations. When presented with ambiguous social situations, children who have experienced trauma are less attentive to relevant social cues and more likely to assume that others have hostile intentions—a pattern known as hostile attribution bias [36, 46–48]. Trauma-exposed children are also more likely to generate aggressive responses to these ambiguous social situations and perceive aggression to be a more effective response than children who have never experienced trauma [36, 46–48].

Together, these social information processing biases prioritize threat-related information in ways that may help children identify early signals of danger in the environment—this rapid and over-identification of threat may help to promote safety in environments where danger is routinely encountered. However, these developmental adaptations following childhood trauma also appear to increase risk for psychopathology transdiagnostically [31, 49]. Perceptual and attentional biases to threat cues are associated with anxiety [50–53], depression [53, 54], PTSD [55–57], psychosis [58–60], and the general psychopathology factor (i.e., p-factor) [61]. Hostile attribution bias, in contrast, is associated with risk for externalizing problems [36, 46, 53] and has also been observed consistently in people with psychosis [62, 63]. Moreover, across many studies, these social information processing biases have been shown to explain the association between childhood trauma and the later emergence of transdiagnostic psychopathology [36, 44, 64].

### Emotional processing mechanisms

A second set of well-established transdiagnostic mechanisms linking childhood trauma with psychopathology involve altered patterns of emotional processing, including heightened emotional reactivity, low emotional awareness, and difficulties with emotion regulation. Consistent with biases in social information processing, youth exposed to trauma exhibit a pattern of emotional responding characterized by increased vigilance and magnified emotional responses to potential threats in the environment. One of the most consistently observed emotional patterns among children with trauma exposure is heightened emotional reactivity, such that salient negative cues in the environment (e.g., angry or fearful faces; social situations depicting people experiencing negative emotions) elicit greater emotional responses in children with trauma histories as compared to children who have never encountered trauma [31, 65]. This heightened emotional reactivity has been observed in studies utilizing behavioral tasks, self-report measures, and experience sampling (i.e., ecological momentary assessment) methods [61, 66–68] as well as neurobiological responses, including greater activation in the amygdala and anterior insula, brain regions that encode emotional salience, to negative relative to neutral stimuli [69–73]. These patterns have been observed inconsistently in children exposed to other forms of adversity, particularly those involving deprivation [73].

In addition to elevated emotional reactivity, childhood trauma exposure also appears to alter patterns of learning about threat in the environment. Young children exposed to trauma exhibit an earlier emergence of aversive learning—as indexed by the ability to generate a conditioned fear response to a previously neutral cue that predicts an aversive stimulus—than children without such exposure [74]. However, by adolescence, trauma-exposed youth exhibit difficulty discriminating between cues that predict threat and safety. For example, during aversive learning, trauma-exposed youth showed less differentiated physiological response between conditioned fear cues and unconditioned safety cues compared to youth without trauma histories [75]. This pattern may reflect a failure to encode the relevant perceptual features of stimuli that predict threat, and contribute to greater generalization of fear responses. The developmental shift from earlier emergence of fear learning to difficulty discriminating between cues that predict threat versus safety may occur when children are raised in environments where violence is experienced chronically and unpredictably—ultimately, making it difficult to learn the most relevant cues for predicting the presence of danger.

Trauma-exposed youth also exhibit a number of difficulties in identifying and regulating their emotions. Childhood trauma is associated with poor emotional awareness—a reduced ability to identify and differentiate one’s own emotions [76]. This tendency for low emotional awareness may contribute to difficulties with emotion regulation, which have also been consistently observed among children exposed to trauma. For example, children exposed to trauma are more likely to report using maladaptive emotion regulation strategies like rumination, expressive suppression, and impulsive responses to distress; greater use of maladaptive emotion regulation strategies has also been observed in studies using behavioral paradigms and caregiver report [31, 61, 67, 77–79]. When asked to use effective emotion regulation strategies like cognitive reappraisal to dampen emotional reactivity, youth exposed to trauma recruit the prefrontal cortex (PFC) to a greater degree than those without trauma histories [71], a pattern that emerges across the transition to adolescence [80]. This pattern suggests that using explicit emotion regulation strategies like cognitive reappraisal might be more difficult or require greater cognitive resources for children who have experienced trauma—potentially as a result of the heightened emotional reactivity common in these youth. These difficulties with emotion regulation have generally not been observed among children exposed to neglect [81].

Beyond these explicit (i.e., intentional) emotion regulation strategies, differences in automatic or implicit forms of emotion regulation have also been observed following childhood trauma. Children exposed to trauma exhibit poor adaptation to emotional conflict [82, 83]—a behavioral process that reflects coupling between the medial prefrontal cortex (mPFC) and amygdala [83]. Activity in the mPFC is associated with dampened amygdala activity in numerous forms of implicit emotion regulation. Consistent with these behavioral patterns, children exposed to trauma also exhibit reduced functional coupling of the amygdala and mPFC in studies of resting-state functional connectivity [84, 85], suggesting that childhood trauma may alter the function of this emotional processing circuit.

These patterns of emotional processing that are common in trauma-exposed children are strongly associated with multiple forms psychopathology. Elevated emotional reactivity is associated with psychopathology transdiagnostically across numerous studies [61, 67, 68, 86]. Recent evidence suggests that low emotional awareness is also associated with the general psychopathology (i.e., p-factor) and mediates the association between childhood trauma and the p-factor [76]. Similarly, disruptions in emotion regulation are associated with virtually all types of psychopathology and predict the onset of internalizing and externalizing problems [87–90] as well as account for comorbidity between disorders [5, 91, 92]. Indeed, elevated emotional reactivity, difficulties with emotion regulation, and reduced functional coupling between the mPFC and amygdala—a neural pattern associated with poor implicit emotion regulation—have all been shown to mediate the link between childhood trauma exposure and transdiagnostic psychopathology [61, 67, 68, 84, 86], including the p-factor [63].

### Accelerated biological aging

A final mechanism through which childhood trauma may confer risk for transdiagnostic psychopathology is accelerated biological aging, whereby exposure to threatening early-life environments might actually alter the pace of development. Life History Theory postulates that experiences in early life can program an individual’s developmental trajectory and pace of aging to respond most effectively to the current environment and environmental demands they are likely to encounter in the future [93–95]. For instance, in a comfortable and predictable environment, a slow and protracted developmental trajectory may be optimal, as it allows for maximal parental investment prior to offspring independence. However, in a harsh or unpredictable environment, a faster pace of development in which children reach adult-like capabilities at an earlier age may be favored in order to maximize reproduction prior to potential mortality.

Two key indicators of accelerated aging in development include pubertal timing and cellular aging. The timing and pace of pubertal development is most commonly measured by age of menarche in females [96, 97] and measures of pubertal stage controlling for chronological age [98, 99]. Cellular aging is most commonly measured by leukocyte telomere length [100, 101] and DNA methylation (DNAm) age [102, 103]. These metrics can be used to evaluate whether the pace of aging at the reproductive or cellular level is occurring faster than what would be expected given an individual’s chronological age.

Numerous studies have found that childhood trauma is associated with earlier pubertal timing [98, 104–106]. A smaller but increasing number of studies have also observed accelerated cellular aging following childhood trauma, including shorter telomere length [107, 108], and advanced DNAm age relative to chronological age [104, 109]. In a recent meta-analysis—including 43 studies of pubertal timing with 114,450 participants and 11 studies of cellular aging with 1560 participants—we found that the association between ELA and accelerated biological development was specific to adversities involving exposure to trauma. Specifically, children exposed to trauma exhibited accelerated biological aging in measures of pubertal development and cellular aging, but children exposed to deprivation (e.g., neglect) and low socioeconomic status did not exhibit this pattern of accelerated aging [110]. These results support dimensional models of adversity arguing that different forms of early-life adversity have unique influences on cognitive, emotional, and neurobiological development [32–34].

Accelerated biological aging is associated with multiple forms of psychopathology. Earlier pubertal timing is associated with elevated levels of risk-taking behavior, delinquency and substance abuse problems [111, 112], depression and anxiety disorders [113, 114], and the p-factor [115] and is considered a transdiagnostic risk factor for psychopathology [116, 117]. Accelerated cellular aging is associated with depression [104, 118], anxiety [119], PTSD [120], psychosis [121–123], and the p-factor [124], suggesting that it may also be a transdiagnostic risk factor. These patterns of accelerated biological aging—including earlier pubertal timing [106] and accelerated cellular aging as indexed by DNAm age [104]—have been shown to mediate the association of trauma exposure with multiple forms of psychopathology. These findings provide strong evidence for accelerated biological aging as a transdiagnostic risk factor for psychopathology following childhood trauma. However, the biological mechanisms through which trauma exposure in childhood actually accelerates reproductive development and cellular aging are not well understood. Characterizing these mechanisms is an important goal for future research.

## Social support as a transdiagnostic protective factor

Children who have experienced trauma often struggle to form and maintain healthy relationships [77, 125, 126]. As such, cultivating social support—a well-established protective factor against the emergence of psychopathology following stressors [127–129]—may be particularly important in this population. Indeed, meta-analysis suggests that children with high levels of social support are less likely to develop trauma-related psychopathology transdiagnostically [130]. In studies that have conceptualized social support broadly, youth who perceive greater social support or engage in more support-seeking behavior following exposure to stress exhibit lower levels of both internalizing and externalizing psychopathology than those who do not [131–135]. Critically, perceived social support is a protective factor that buffers against the onset and progression of psychopathology following trauma exposure [136–138].

Caregiver support is a particularly important form of social support that may protect against the emergence of psychopathology following childhood trauma. Recent evidence suggests that the presence of a supportive caregiver can buffer against the elevated threat-related processing that is common in children who have experienced trauma. The presence of a supportive caregiver is associated with lower amygdala reactivity, greater functional coupling of the mPFC and amygdala during threat processing, and enhanced discrimination of threat and safety cues during aversive learning [139, 140]. Thus, caregiver buffering of threat-related processing may be one particularly important pathway through which support can mitigate risk for psychopathology in children who have experienced trauma. Future work should examine whether interventions that promote caregiver support and broader social support-seeking behaviors may have potential for reducing vulnerability to psychopathology among children exposed to trauma.

## Intervention implications

Understanding the mechanisms that contribute to multiple forms of psychopathology can facilitate identification of the most important targets for intervention. In contrast to disorder-specific approaches that have historically been common, altering these transdiagnostic mechanisms through intervention provides the opportunity to more broadly reduce psychopathology risk. The clear advantages of such an approach have led to the emergence of transdiagnostic treatments for both children and adults [141, 142].

With regard to childhood trauma, the transdiagnostic mechanisms reviewed here—particularly in the domains of social information and emotional processing—reflect characteristics that could be targeted with early interventions aimed at preventing the onset of psychopathology. Elsewhere, we articulate how these social and emotional processing mechanisms could be targeted with existing evidence-based intervention techniques to prevent child and adolescent psychopathology [143]. Although specific techniques that could reduce the accelerated pace of biological aging following childhood trauma are less well understood, mindfulness interventions have shown promise for slowing the pace of biological aging in adults [144]. Moreover, early pubertal maturation is a readily observable feature that could allow medical practitioners to identify youth who may require additional assessment for childhood trauma exposure and early intervention within the context of routine primary care visits. Determining if early interventions targeting these mechanisms have the potential to reduce risk for psychopathology, and whether the effectiveness of such approaches vary for children in different stages of development, is a critical question for future research.

## Conclusion

Transdiagnostic processes confer risk for multiple types of psychopathology, explain comorbidity of different disorders, and provide targets for early intervention and treatment. Childhood trauma exposure is a powerful transdiagnostic factor that is associated with elevated risk for virtually all commonly occurring forms of psychopathology. Children who experience trauma exhibit consistent changes in social information processing in ways that involve the prioritization and over-identification of threat in the environment; patterns of emotional processing characterized by elevated emotional reactivity, low emotional awareness, and difficulties with emotional learning and emotion regulation; and accelerated biological aging across metrics of pubertal development and cellular aging. Together, these patterns reflect developmental adaptations to an early environment characterized by danger that may provide short-term benefits. However, these changes in social information processing, emotional responding, and the pace of biological aging are also consistently associated with elevated risk for the emergence of multiple forms of internalizing and externalizing psychopathology and explain the link between childhood trauma exposure and transdiagnostic psychopathology. As such, these mechanisms represent critical targets for early interventions aimed at preventing the emergence of psychopathology in children who have experienced trauma.

## Data Availability

Not applicable.
